# Identification of AgRP cells in the murine hindbrain that drive feeding

**DOI:** 10.1016/j.molmet.2024.101886

**Published:** 2024-01-19

**Authors:** Tomas P. Bachor, Eunsang Hwang, Ernie Yulyaningsih, Kush Attal, Francois Mifsud, Viana Pham, Eirini Vagena, Renzo Huarcaya, Martin Valdearcos, Christian Vaisse, Kevin W. Williams, Paul J. Emmerson, Allison W. Xu

**Affiliations:** 1 Diabetes Center and Department of Anatomy, University of California, San Francisco, California, USA; 2 Center for Hypothalamic Research, Department of Internal Medicine, the University of Texas Southwestern Medical Center at Dallas, Dallas, TX, USA; 3 Lilly Research Laboratories, Lilly Corporate Center, Eli Lilly & Company, Indianapolis, IN, USA

**Keywords:** AgRP, Hindbrain, Feeding

## Abstract

**Objective:**

The central melanocortin system is essential for the regulation of food intake and body weight. Agouti-related protein (AgRP) is the sole orexigenic component of the central melanocortin system and is conserved across mammalian species. AgRP is currently known to be expressed exclusively in the mediobasal hypothalamus, and hypothalamic AgRP-expressing neurons are essential for feeding. Here we characterized a previously unknown population of AgRP cells in the mouse hindbrain.

**Methods:**

Expression of AgRP in the hindbrain was investigated using gene expression analysis, single-cell RNA sequencing, immunofluorescent analysis and multiple transgenic mice with reporter expressions. Activation of AgRP neurons was achieved by Designer Receptors Exclusively Activated by Designer Drugs (DREADD) and by transcranial focal photo-stimulation using a step-function opsin with ultra-high light sensitivity (SOUL).

**Results:**

AgRP expressing cells were present in the area postrema (AP) and the adjacent subpostrema area (SubP) and commissural nucleus of the solitary tract (cNTS) of the mouse hindbrain (termed AgRP^Hind^ herein). AgRP^Hind^ cells consisted of locally projecting neurons as well as tanycyte-like cells. Food deprivation stimulated hindbrain *Agrp* expression as well as neuronal activity of subsets of AgRP^Hind^ cells. In adult mice that lacked hypothalamic AgRP neurons, chemogenetic activation of AgRP neurons resulted in hyperphagia and weight gain. In addition, transcranial focal photo-stimulation of hindbrain AgRP cells increased food intake in adult mice with or without hypothalamic AgRP neurons.

**Conclusions:**

Our study indicates that the central melanocortin system in the hindbrain possesses an orexigenic component, and that AgRP^Hind^ neurons stimulate feeding independently of hypothalamic AgRP neurons.

## Highlights

• AgRP is expressed in the dorsal vagal complex of the mouse hindbrain.
• Food deprivation increases expression of AgRP in the hindbrain and neuronal activity of hindbrain AgRP cells.
• Activation of hindbrain AgRP cells stimulates feeding, and this action is independent of hypothalamic AgRP neurons.

## Graphical abstract

**Figure:**
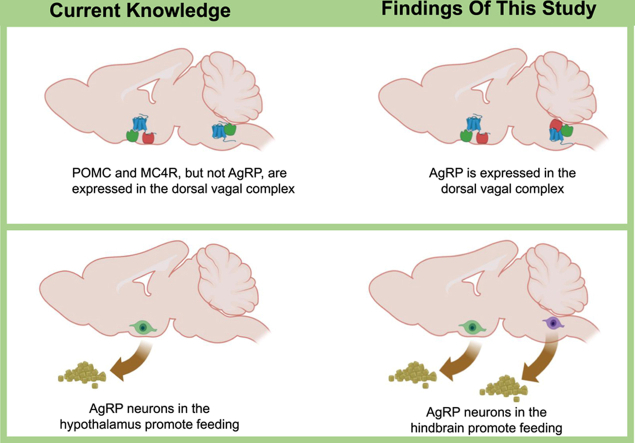

## 1 Introduction

The central melanocortin system is composed of proopiomelanocortin (POMC), agouti-related protein (AgRP), melanocortin-3 receptor (MC3R) and melanocortin-4 receptor (MC4R). Alpha-melanocyte-stimulating hormone, produced by the POMC neurons, is the agonist of MC3R and MC4R, whereas AgRP is an antagonist and inverse agonist of these receptors. Loss of function of POMC, MC3R or MC4R in mice leads to increased fat mass [1]. Consistent with the orexigenic function of AgRP, infusion of AgRP into the brain or transgenic overexpression of *Agrp* in mice leads to obesity [2,3]. The arcuate nucleus (ARC) of the hypothalamus is a major brain region where POMC and AgRP neurons reside, and these neurons project widely to multiple forebrain and hindbrain regions. ARC AgRP neurons (AgRP^ARC^ neurons) are GABAergic and they co-express neuropeptide Y (NPY), a potent orexigenic peptide. Optogenetic or chemogenetic stimulation of AgRP^ARC^ neurons leads to intense feeding [4,5]. Thus, AgRP represents the sole orexigenic component of the central melanocortin system, and AgRP^ARC^ neurons are key drivers of feeding.

Notably, in addition to being present in the hypothalamus, POMC and MC4R-expressing neurons are also found in the dorsal vagal complex (DVC) of the caudal brainstem. The DVC is composed of the area postrema (AP), the nucleus of the solitary tract (NTS), and the dorsal motor nucleus of the vagus nerve (DMV). The AP is a circumventricular organ and lacks an intact blood–brain barrier (BBB). Thus, cells in the AP can communicate directly with circulating signals. The AP, NTS and DMV express receptors for a myriad of circulating satiety signals such as amylin, insulin, CCK, and GLP-1, and are sites of integration for ascending neural signals coming from the gastrointestinal system [6]. MC4R is abundantly expressed in the DMV, and these neurons mediate a variety of autonomic functions [7,8]. On the other hand, POMC is expressed in the commissural NTS (cNTS) [[9], [10], [11]]; Chemogenetic activation of NTS POMC neurons inhibits feeding [12], indicating that they are anorexigenic. POMC neurons in the NTS project to other brainstem areas as well as to the lateral parabrachial nucleus, hypothalamus and nucleus accumbens [13]. AgRP neurons are currently known to reside only in the ARC of the hypothalamus [14]. As such, the hindbrain melanocortin system is unbalanced as AgRP, the orexigenic component of this system, has not been identified in this region. In this study, we describe a previously unknown population of AgRP-expressing cells in the AP and adjacent subpostrema area (SubP) and cNTS, and demonstrate that these cells were orexigenic in function.

## 2 Material and methods

### 2.1 Mice

Mice were housed in a barrier facility with 12-hour light–dark cycles (7 am–7 pm) and fed a standard mouse chow (LabDiet #5053). *Agrp*^*−/−*^ mice [15] were provided by Dr. Greg Barsh. *Rosa*^*LSL-SOUL*^ mice [16] were provided by Dr. Guoping Feng. *Agrp*^*CreERt2*^ [17] were provided by Dr. Joel Elmquist. *Agrp*^*Cre*^ (also called *Agrp*^*IRES-Cre*^) [18] (*Agrp*^*tm1(cre)Lowl*^*/J*, JAX stock # 012,899)*, Rosa*^*LSL-Gq-DREADD*^ [19] (JAX stock# 026,220), *Mc4r*^*Cre*^ [20](JAX stock# 030,759), and *Rosa*^*LSL-tdT*^ (Ai14 Cre reporter) [21] mice (JAX stock# 007914) were obtained from the Jackson Laboratory. All *Agrp*^*IRES-Cre*^ mice were screened for any widespread ectopic recombination event and such mice were excluded. Male (6- to 18-week-old) pathogen-free mice were used for all experiments. For electrophysiological studies, *Agrp*^*IRES-Cre*^, *Rosa*^*LSL-tdT*^ mice were maintained at University of Texas Southwestern Medical Center under standard laboratory conditions (12 h on/off; lights on at 7:00 a.m.) and a temperature-controlled environment with food and water available ad libitum. Animal care and all experiments were approved by the Institutional Animal Care and Use Committees at University of California at San Francisco and University of Texas Southwestern.

### 2.2 Single cell RNA sequencing and analysis

To identify the area postrema (AP) during microdissection, 50 μl of a 1 % Evans Blue solution was administered transcardially to a male P10 mouse previously anesthetized with intraperitoneal injection of Avertin (250 mg/kg – Sigma–Aldrich #T48402). After 5 min, the mouse was decapitated, and the brain was dissected and placed into a petri dish with ice cold sterile 1X PBS under a dissecting microscope. After visually identifying the AP (in blue), the AP-centric region was microdissected and immediately dissociated into a single cell suspension. The dissociation was carried out according to Saxena et al. [22] with volumes adjusted for tissue size. Briefly, the tissue was triturated and incubated with Papain-DNase1 solution using the Worthington Papain Dissociation System (#LK003150, Worthington, NJ) for 60 min at 37 °C with constant agitation. All components of the dissociation system were equilibrated with 95 % O2, 5 % CO_2_ before use. The tissue was then further triturated by pipetting until a homogenous cell suspension was observed. The suspension was then centrifugated and resuspended in 0.04 % BSA. Cell count and viability was assessed with 0.4 % Trypan Blue staining and an automatic cell counter (Countess™ II). Single cell suspension was submitted to the UCSF Genomics Core for chromium single-cell droplet generation using the 10X Genomics platform, library preparation and sequencing.

For data analysis, raw RNA expression matrices were created for all 10X sequencing runs. All cells (n = 5,706) were merged into one matrix. Using Seurat software (version 4.0.6) [23], low-quality cells containing <200 detected genes and cells with a mitochondrial RNA content >5 % were filtered out. In addition, only genes with expression in >3 cells were retained for downstream analysis (n = 1,851). Gene expression in each cell was then normalized by the cell's total expression. Afterwards, Seurat software we used to perform clustering. The 2,000 most variable genes in the dataset were identified for Principal Component Analysis (PCA). After scaling the dataset and centering the data along each variable gene, PCA was performed on the scaled data. To determine which principal components (PCs) to use for clustering, the cumulative variance accounted for by each subsequent PC was plotted. The first 20 PCs were chosen for further clustering analysis and input for Uniform Manifold Approximation and Projection (UMAP) clustering. Using the default parameters in Seurat to construct an embedding that places similar cells together in 2-dimensional space, similar cell types and clusters were identified using the FindClusters function in Seurat, setting the resolution to 0.02 to identify 8 cell types. Afterwards, the RunUMAP function returned a visual, two-dimensional plot of these cell clusters, with cells expressing similar gene expressions within the variable gene set localizing near each other.

### 2.3 Immunostaining procedure

Anesthetized mice were perfused with 4 % (w/v) paraformaldehyde. Brains were post-fixed in 4 % paraformaldehyde overnight, immersed in 30 % sucrose overnight, and cryosectioned at 20 or 35 μm. Immunohistochemistry was performed with goat anti-AgRP (1:500, Neuromics #GT15023), rabbit anti-DsRed (1:500, Rockland #600-401-379), rabbit anti-FOS (1:1000, Cell Signaling #9F6), Mouse anti HuC/D (1:500, ThermoFisher #16A11) and Mouse anti-Nestin (1:200, Millipore Sigma #MAB353). For FOS staining, sections were incubated in base (1 % NaOH with 1 % H2O2), 0.3 % glycine, and 0.3 % SDS unmasking solutions in 1x PBS for 10 min. Sections were incubated in a blocking solution then with the primary antibody overnight at 4 °C and washed. A solution of secondary antibodies was applied for 2 h at room temperature. For FOS staining by DAB (3,3′-diaminobenzidine), VECTASTAIN elite ABC peroxidase kit (Vector Laboratories), and the ImmPACT DAB peroxidase substrate (Vector Laboratories) were used. Immunofluorescent images were acquired with an Olympus BX51WI microscope equipped with a QImaging Retiga 2000R digital camera or a Leica SP8 confocal microscope.

### 2.4 Brain slice preparation

Brain slices were prepared from young adult male mice (6–18 weeks old) as previously described [[24], [25], [26]]. Briefly, male mice were deeply anesthetized with i.p. injection of 7 % chloral hydrate and transcardially perfused with a modified ice-cold ACSF (described below). The mice were then decapitated, and the entire brain was removed and immediately submerged in ice-cold, carbogen-saturated (95 % O2 and 5 % CO2) ACSF (126 mM NaCl, 2.8 mM KCl, 1.2 mM MgCl2, 2.5 mM CaCl2, 1.25 mM NaH2PO4, 26 mM NaHCO3, and 5 mM glucose). Coronal sections (250 mm) were cut with a Leica VT1000S Vibratome and then incubated in oxygenated ACSF (32 °C–34 °C) for at least 1 h before recording. The slices were bathed in oxygenated ACSF (32 °C–34 °C) at a flow rate of ∼2 ml/min. All electrophysiology recordings were performed at room temperature.

### 2.5 Whole-cell recordings

The pipette solution for whole-cell recording was modified to include an intracellular dye (Alexa Fluor 350 hydrazide dye) for whole-cell recording: 120 mM K-gluconate, 10 mM KCl, 10 mM HEPES, 5 mM EGTA, 1 mM CaCl2, 1 mM MgCl2, and 2 mM MgATP, 0.03 mM Alexa Fluor 350 hydrazide dye (pH 7.3). Epifluorescence was briefly used to target fluorescent cells, at which time the light source was switched to infrared differential interference contrast imaging to obtain the whole-cell recording (Zeiss Axioskop FS2 Plus equipped with a fixed stage and a QuantEM:512SC electron-multiplying charge-coupled device camera). Electrophysiological signals were recorded using an Axopatch 700B amplifier (Molecular Devices); low-pass filtered at 2–5 kHz, and analyzed offline on a PC with pCLAMP programs (Molecular Devices). Membrane potentials and firing rates were measured from AgRP neurons in brain slices. Recording electrodes had resistances of 2.5–5 MΩ when filled with the K-gluconate internal solution.

### 2.6 Neonatal MSG injection

In order to ablate hypothalamic AgRP neurons, newborn mice were given a single subcutaneous injection of monosodium glutamate (MSG) at postnatal day 2, similarly to previously reported [27]. MSG (#G1251, Millipore-Sigma) was prepared at a concentration of 0.35 g/ml in UltraPure™ DNase/RNase-Free Distilled Water (Invitrogen). All solutions were prepared on the day of injection and filter sterilized. MSG was injected subcutaneously in a volume of 10 ul/g body weight at a dose 3.5 g/kg.

### 2.7 Cannula implantation and transcranial photo-stimulation

For transcranial photo-stimulation, 7–10 week-old male mice were deeply anesthetized with a combination of ketamine and xylazine and supplemented with isoflurane for the duration of the procedure. Animals were placed on a heating pad and the skull was immobilized in a stereotaxic apparatus (Model 1900, Stereotaxic Alignment Systems, 1 μm resolution, David Kopf Instruments). A custom-made ceramic mono fiber-optic cannula without the protruding fiber optics (400 μm, NA 0.37, 0 mm fiber length; Doric, Canada) was attached to the surface of the intact skull at −7.50 mm from bregma. Two anchoring screws (Stoelting Co) and black dental cement (Lang Dental Manufacturing Co., Inc.) was applied to the surroundings to secure and cover the cannula. Mice were allowed to recover for at least 7 days before experiments. At the time of experiment, mice were placed in a room with controlled light below 700 lux and were allowed to acclimate for 30 min before stimulation. Basal food intake was established 90 min before stimulation. Mice were then placed on an empty cage, and a rotary joint patch cable with Ø400 μm fiber and Ø1.25 mm ferrule (ThorLabs) was securely attached to the cannula through a ceramic mating sleeve covered with a black shrinking tube to prevent optical noise. Animals were allowed to move freely for 10 min. Laser light was then applied for 5 min (473 nm DPSS, 50 mW; Laser Glow Techologies). After, animals were placed in another cage where food intake was measured 90 min after photo stimulation.

### 2.8 Indirect calorimetry measurements

Metabolic studies were performed as previously described [[27], [28], [29]]. In brief, during metabolic monitoring, mice were singly housed for 3 days before measurements were taken and allowed one day to acclimatize in CLAMS chambers (Oxymax®-CLAMS; Columbus Instruments.). Food intake, O_2_ consumption, CO_2_ production, energy expenditure, RER, and locomotor activities were analyzed.

### 2.9 CNO and tamoxifen administration

CNO (Clozapine N-oxide, C0832; Sigma–Aldrich) powder was dissolved in DMSO and then diluted in sterile saline solution (0.9 % NaCl). Both *Agrp*^*hM3Dq*^ mice and the control mice were first administered with saline i.p. to control for the stress of injection. After that, both groups of mice were given CNO (0.5 mg/kg, i.p.) to control for potential metabolic effects of CNO. Tamoxifen (#T5648; Siga-Aldrich) was dissolved in corn oil and filter-sterilized. 4-week-old *Agrp*^*CreERT2*^ mice received one dose of TAM (10 μl/g) by oral gavage per day for 3 days and were perfused 4 days after last oral gavage.

### 2.10 Statistical analysis

Unpaired two-tailed Student's *t*-test was used to compare two independent groups. When two genotypes and multiple treatments/conditions were compared, repeated-measures two-way ANOVA with multiple comparisons were used. Statistical analysis was performed using GraphPad Prism 9.0 software (GraphPad Software, Inc, La Jolla, CA, USA).

## 3 Results

### 3.1 *Agrp* is expressed in the mouse hindbrain

**Figure 1:**
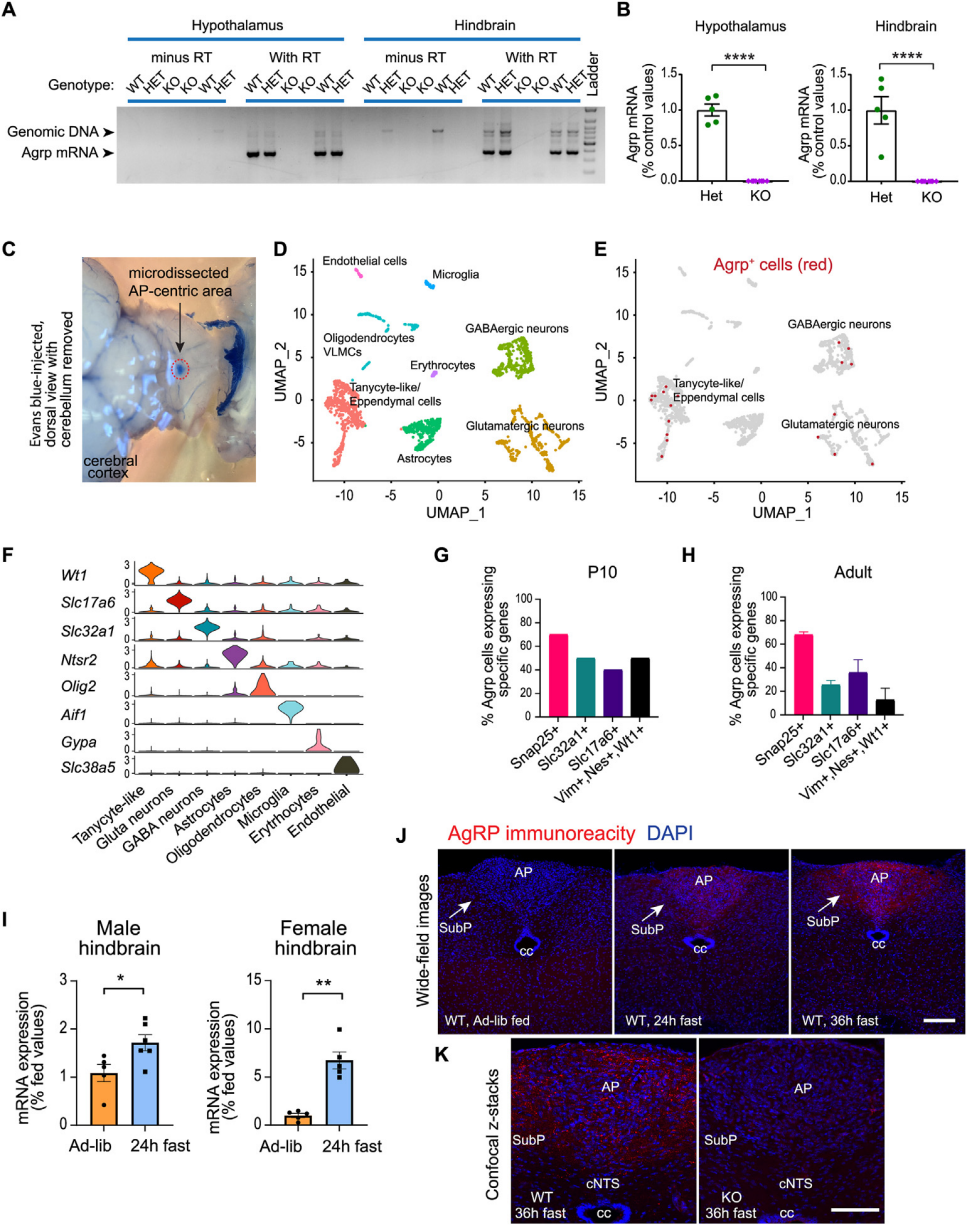
**AgRP is expressed in the mouse hindbrain and its expression increases with food deprivation.** (**A**) RT-PCR analysis of *Agrp* mRNA expression in adult mice. PCR products were fractionated on agarose gel. PCR product from cDNA is 378 bp whereas PCR product from genomic DNA is 715 bp. WT: *Agrp*^*+/+*^; Het: *Agrp*^*+/−*^, KO: *Agrp*^*−/−*^ mice. (**B**) qRT-PCR analysis of *Agrp* mRNA expression in adult mice heterozygous (Het) or null (KO) for AgRP. (**C**) Picture of the AP marked by Evans blue accumulation and area of microdissection. Cerebellum was removed to expose the AP. (**D-E**) scRNAseq analyses of the microdissected AP tissues of a P10 mouse, showing cluster plot in D and *Agrp*-expressing cells in E. (**F**) Violin plot showing expression of signature genes that mark each cell cluster. (**G**) scRNAseq analysis of marker genes for neurons, GABAergic and glutamatergic neurons and tanycytes in *Agrp* cells in microdissected AP-centric tissue from a P10 mouse. (**H**) snRNAseq analysis of *Agrp* cells from microdissected AP tissues of chow-fed adult mice [30] (Geo accession number GSE160938). Data were mean of two replicates; each were pools of multiple mice. (**I**) *Agrp* mRNA expression in the hindbrain of male and female mice that were *ad-lib* fed or fasted for 24h. (**J**) Wide-field images of immunofluorescence analysis with AgRP antibody showing AgRP-positive boutons in mice under fed, 24h fasting or 36h fasting conditions. Scale bar: 100 μm. (**K**) Confocal Z-scan showing presence of AgRP-positive boutons in the AP of 36h-fasted wild-type (WT) mice but not in AgRP-deficient (KO) mice. Scale bar: 100 μm ∗p < 0.05, ∗∗p < 0.01, ∗∗∗∗p < 0.0001 by students' t-test. AP: area postrema. SubP: subpostrema area. cc: central canal. cNTS: commissural nucleus of solitary tract.

Since *Pomc* is expressed in the DVC of the hindbrain, we explored if AgRP is also expressed in this brain region. Hypothalamus and hindbrain tissues were harvested from adult *Agrp*^*+/+*^, *Agrp*^*+/−*^ and *Agrp*^*−/−*^ mice. RT-PCRs were carried out using forward and reverse primers spanning coding Exon 4 and Exon 6 respectively. PCR fragments corresponding to the size of the *Agrp* mRNA was amplified from both hypothalamus and the hindbrain, but not from samples where reverse transcriptase was omitted (Figure 1A). Sequencing of the PCR products confirmed their identities as AgRP transcripts. No RT-PCR product was amplified from either the hypothalamus or the hindbrain of the *Agrp*^*−/−*^ mice (Figure 1A). In addition, real time RT-PCR analysis showed that *Agrp* mRNA were expressed in the hindbrain of *Agrp*^*+/−*^ but not in *Agrp*^*−/−*^ mice (Figure 1B), and that *Agrp* expression in the hindbrain was lower than that in the hypothalamus (Fig. S1).

To explore if AgRP-expressing cells are located near the DVC of the mouse hindbrain, we subjected cells isolated from the AP region of postnatal day 10 (P10) mouse to single-cell RNA-sequencing (scRNAseq). Since the AP is a circumventricular organ that is outside the BBB, evans blue, a colored dye that does not penetrate the BBB and accumulates in circumventricular organs, was injected peripherally so that the AP could be visualized with the dye and microdissected under a dissecting microscope (Figure 1C). The microdissected tissue was dissociated into a single-cell suspension and subjected to scRNAseq using the 10X Genomics platform. *Agrp* mRNA expressing cells were present in the dataset, and they clustered with GABAergic neurons, glutamatergic neurons, and tanycyte-like/ependymal cells (Figure 1D–F). Accordingly, subsets of AgRP cells expressed pan-neuronal marker *Snap25*, GABAergic neuronal marker *Slc32a1,* glutamatergic neuronal marker *Slc17a6*, or tanycyte marker *Vimentin*, *Nestin* and *Wt1* (Figure 1G). To gain insight into AgRP cells in the AP of adult mice, we analyzed the publicly available single-nuclei RNAseq (snRNAseq) dataset, which contains pooled micro-dissected AP tissues from adult mice (Geo accession number GSE160938) [30]. *Agrp*-expressing cells were present in adult AP tissue, and majority of the cells expressed *Snap25* with mixed populations of *Slc32a1* and *Slc17a6*-expressing cells (Figure 1H). Moreover, AgRP-expressing cells in the hindbrain show specific gene expression profiles comparing to other cells (Fig. S2). Together, these results indicate that *Agrp* expressing cells are present in the mouse hindbrain.

### 3.2 Hindbrain AgRP expression is low in adult mice but increased after fasting

One hallmark feature of hypothalamic AgRP is its marked and progressive increase in expression following food deprivation [31]. Despite identification of *Agrp-*expressing cells by scRNAseq, AgRP immunoreactivity was below detection in the hindbrain of adult mice under *ad-lib* fed condition. However, hindbrain *Agrp* mRNA expression was stimulated by fasting in both male and female mice (Figure 1I). AgRP immunoreactivity, while not detectable in *ad-lib* fed mice, became detectable in the AP, SubP, and cNTS after fasting in wild-type (Figure 1J) but not in AgRP-deficient mice (Figure 1K). This result suggests that hindbrain AgRP is responsive to energy deficit.

### 3.3 Hindbrain AgRP-expressing cells are located in the AP, SubP and cNTS

**Figure 2:**
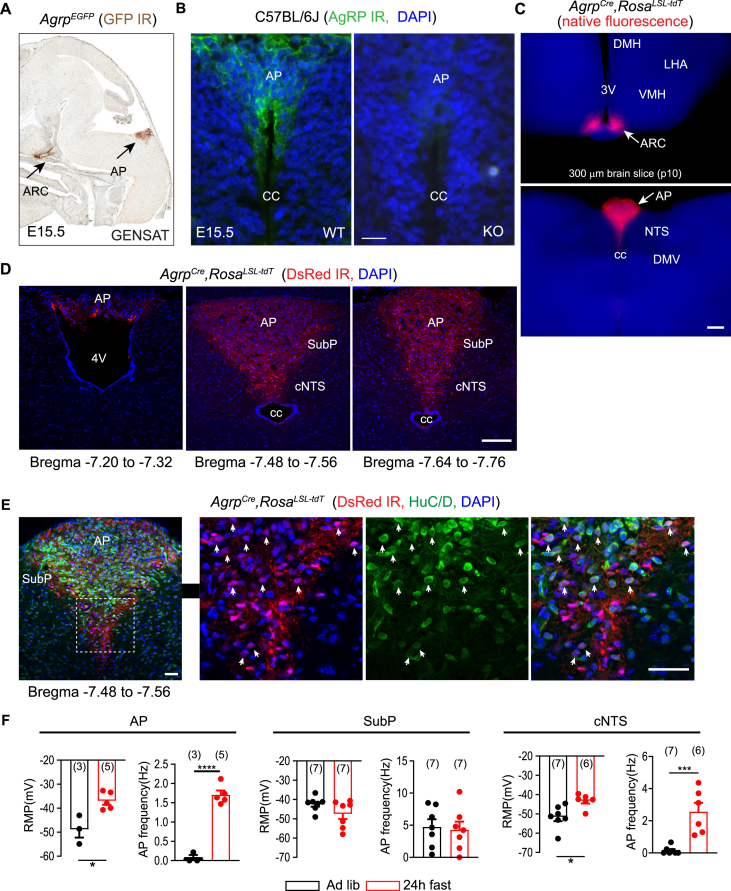
**AgRP-expressing cells are present in the AP and cNTS of the mouse hindbrain and their firing rates increase with food deprivation.** (**A**) E15.5 mouse embryos expressing EGFP under the control of AgRP promoter showing expression in the ARC and AP (image ID 78824 is cited with permission from GENSAT). (**B**) AgRP immunoreactivity in the developing AP at E15.5 in wild-type (WT) and AgRP-deficient (KO) mice. Scale bar: 20 μm. (**C**) Native fluorescence signals in 300 μm fresh vibratome sections from 10-day-old *Agrp*^*Cre*^*, Rosa*^*TdT/+*^ mice. Scale bar: 200 μm. (**D**) Immunofluorescence analysis with DsRed antibody showing tdTomato expression from rostral to caudal AP in adult *Agrp*^*Cre*^*, Rosa*^*TdT/+*^ mice. Scale bar: 100 μm. (**E**) Single-scan confocal microscopy of immunoreactivity of neuronal marker HuC/D and tdT in the hindbrain of 9-week-old *Agrp*^*Cre*^*, Rosa*^*TdT/+*^ mice. Magnified views of the box area in left panel are shown in the 3 panels on the right. Scale bar: 50 μm. (**F**) Histograms depicting changes in the resting membrane potential (RMP) and action potential frequency (APF) of tdTomato^+^ neurons in the AP, SubP, and cNTS from ad libitum fed and 24 h fasted *Agrp*^*Cre*^*, Rosa*^*TdT/+*^ mice. Data are expressed as mean ± SEM. ∗p < 0.05, ∗∗∗p < 0.001, ∗∗∗∗p < 0.0001, determined by unpaired t-test." ARC: arcuate nucleus. DMH: dorsomedial hypothalamus. LHA: lateral hypothalamic area. AP: area postrema. SubP: subpostrema area. cNTS: commissural nucleus of solitary tract. 3V: third ventricle. 4V: fourth ventricle. cc: central canal.

To evaluate if reporter expression is in hindbrain AgRP cells, we examined the distribution of *Agrp*^*EGFP*^ in transgenic mice from the GENSAT Brain Atlas, where EGFP was expressed under the control of AgRP promoter from a bacterial artificial chromosome. EGFP expression was present in the AP of E15.5 mouse embryos (Figure 2A) but reduced in postnatal P7 pups and adult mice (GENSAT Brain Atlas). Consistent with these data, we detected AgRP immunoreactivity in the developing AP of E15.5 WT but not AgRP-deficient embryos (Figure 2B).

**Figure 3:**
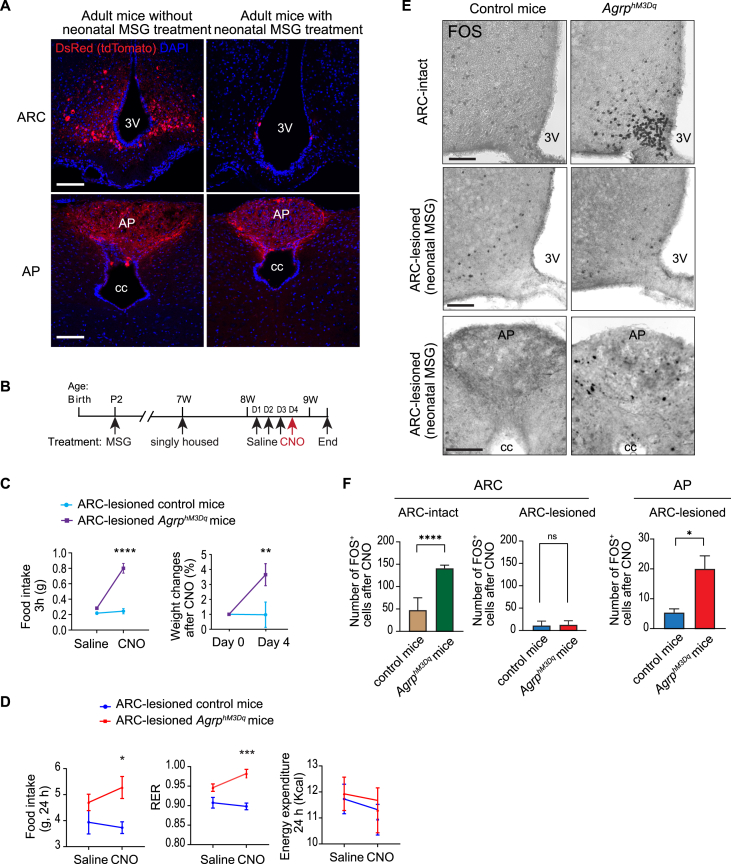
**DREADD-mediated activation of AgRP cells in the hindbrain promotes feeding. (A)** Mice carrying *Agrp*^*Cre*^*, Rosa*^*LSL-tdT*^ were injected subcutaneously with MSG (3.5 mg/kg) or vehicle at postnatal day 2 (P2) and examined in adulthood. tdTomato^+^ cells were visualized with DsRed immunoreactivity, followed by single scan confocal microscopy. 3V: third ventricle. ARC: arcuate nucleus. AP: area postrema. Scale bar: 100 μm. (**B–C**) Mice expressing hM3Dq-DREADD(Gq-DREADD) in AgRP cells (*Agrp*^*Cre*^*;Rosa*^*LSL-hM3Dq*^*, termed Agrp*^*hM3Dq*^) or control mice (*Agrp*^*Cre*^ or *Rosa*^*LSL-hM3Dq*^) were injected with MSG (3.5 mg/kg, s.c.) at P2 to ablate ARC AgRP neurons (ARC-lesioned). n = 6–8 per group. When these mice reached young adulthood (8-week-old), saline was injected on 3 consecutive days, followed by a single CNO (0.5 mg/kg, i.p.) injection. Food intake and body weight were measured at the indicated time periods. (**D**) ARC-lesioned *Agrp*^*hM3Dq/+*^ and control mice were injected with saline and CNO while in the CLAMS chambers. Food intake, RER and energy expenditure were measured. n = 3–4 per group. (**E-F**) ARC-intact and ARC-lesioned *Agrp*^*hM3Dq/+*^ and control mice were treated with CNO, and processed for FOS immunoreactivity 90 min later. Numbers of FOS-positive cells in the ARC and AP were quantified. Scale bar: 100 μm. Data are mean ± SEM. ∗p < 0.05, ∗∗p < 0.01, ∗∗∗p < 0.001, ∗∗∗∗p < 0.0001 by 2-WAY ANOVA with repeated measures and multiple comparisons (panel C and D), 2-WAY ANOVA or student's *t*-test (panel F).

We next evaluated whether mice with Cre recombinase knocked into the *Agrp* locus (*Agrp*^*Cre*^, also named *Agrp*^*IRES-Cre*^) [18] and *Rosa*^*LSL-tdT*^ would direct Cre-dependent tdTomato (tdT) expression in the hindbrain. Strong tdTomato fluorescence was detected in the ARC of the hypothalamus from freshy cut 300 μm brain slices from P10 *Agrp*^*Cre*^,*Rosa*^*LSL-tdT*^ mice (Figure 2C). In contrast, in the same slices, dorsomedial hypothalamus (DMH) and lateral hypothalamic area (LHA), regions that are known to be innervated by AgRP^ARC^ neurons, were negative for tdTomato signal (Figure 2C), suggesting that tdTomato signal mostly originates from AgRP^ARC^ neuronal cell bodies in these postnatal mice. Notably, AP region in the hindbrain also exhibited strong direct tdTomato fluorescence (Figure 2C). By immunofluorescence analysis against tdTomato protein and confocal microscopy, tdTomato expression was detected from the rostral to caudal AP in adult mice under normal fed condition. tdTomato signal was also found in SubP and cNTS (Figure 2D). tdTomato^+^ fibers were confined to AP, SubP and cNTS, suggesting that these cells project locally. Consistent with our findings, tdTomato mRNA is present in the AP of adult mice expressing the *Agrp*^*Cre*^*; Rosa*^*LSL-tdT*^ (Figure 3, image cited from Allen Brain Atlas**).** In addition, we examined *Agrp*^*CreERt2*^; *Rosa*^*LSL-tdT*^ mice, in which expression of CreERt2 is directed by the *Agrp* regulatory elements on a bacterial artificial chromosome [17]. By confocal microscopy, tdTomato expression was observed in the AP, SubP and cNTS of these mice (Fig. S4). By double immunofluorescence assay, about 58 % tdTomato-positive cell bodies were found to co-express neuronal marker HuC/D, suggesting that they are neurons (Figure 2E). A subset of tdTomato-positive cells was positive for nestin, a gene that is restricted to tanycytes and neural progenitors in the adult brain (Fig. S5). Collectively, by using RT-PCR, scRNAseq, and several independent transgenic lines, we show that AgRP was expressed in the DVC of the caudal brainstem.

In *Agrp*^*Cre*^*; Rosa*^*LSL-tdT*^ mice, AgRP-immunoreactive boutons were present in the AP, Sub and cNTS area, and they were partially colocalized with tdTomato expression (Fig. S6). AgRP protein is typically detected only in axonal boutons whereas tdTomato protein is also located in other subcellular processes such as projections. tdTomato may also mark descendant cells that are derived from embryonic AgRP cells but with AgRP expression quenched or reduced in adulthood. Therefore, the tdTomato-positive but AgRP-negative signals likely represent a combination of the above scenarios. The expressions of reporters in the hindbrain of 3 independent transgenic lines, i.e. *Agrp*^*EGFP*^*, Agrp*^*Cre*^*, Agrp*^*CreERt2*^, support the notion that the reporter-expression is specific to AgRP-expressing cells and their lineages.

### 3.4 Food deprivation stimulates activity of *Agrp*^*Cre*^*::tdTomato*-positive neurons in the AP and NTS

We next investigated if energy deficit may influence the activity of tdTomato^+^ neurons in the AP, SubP and cNTS of *Agrp*^*Cre*^,*Rosa*^*LSL-tdT*^ mice by conducting whole-cell patch clamp electrophysiological recordings. Following a 24 h fast, tdTomato^+^ neurons in both AP and cNTS areas exhibited a depolarized resting membrane potential (RMP) and an increased action potential frequency (APF) (AP: RMP -36.9 ± 1.7 mV, APF 1.7 ± 0.1 Hz; cNTS: RMP -43.2 ± 1.5 mV, APF 2.6 ± 0.6 Hz) when compared to ad-libitum mice (AP: RMP: 48.8 ± 3.4 mV, APF: 0.1 ± 0.1 Hz; cNTS: RMP: 51.6 ± 2.2 mV, APF: 0.1 ± 0.1 Hz) (Figure 2F). However, while tdTomato^+^ neurons in the SubP area exhibited a trend towards a hyperpolarized membrane potential following food deprivation, this did not reach statistical significance (ad-libitum: 42.3 ± 1.4 mV; 24h fasting: 47.5 ± 2.7 mV) (Figure 2F). Additionally, the APF in SubP AgRP-expressing neurons remained unchanged following fasting (ad-libitum: 4.7 ± 1.2 Hz; 24h fasting: 4.3 ± 1.3 Hz) (Figure 2F). Thus, food deprivation activated the tdTomato^+^ neurons in the AP and cNTS, suggesting that AgRP neurons in the hindbrain are able to sense energy deficit. The lack of response of cells in the SubP area to fasting indicates region-specific heterogeneity among hindbrain AgRP neurons.

### 3.5 Chemogenetic activation of AgRP cells in mice lacking ARC AgRP neurons results in hyperphagia and weight gain

We subsequently examined if activation of AgRP cells in mice lacking hypothalamic AgRP neurons would lead to increased food intake. Monosodium glutamate (MSG), an excitotoxic neurotoxin, is known to rapidly lesion the arcuate nucleus [[32], [33], [34], [35]]. We also show that injecting neonatal mice with MSG eliminates 99 % of all ARC AgRP/NPY neurons in these mice when they reach adulthood [27]. By examining adult *Agrp*^*Cre*^*; Rosa*^*LSL-tdT*^ mice that were injected subcutaneously with MSG or vehicle at P2, ARC tdTomato cells were ablated as expected, but tdTomato cells in the AP were not reduced (Figure 3A), enabling us to study this subpopulation of cells without the interference from AgRP^ARC^ neurons.

Mice harboring Cre-activatable DREADD (*Rosa*^*LSL-hM3Dq*^) were crossed with mice carrying *Agrp*^*Cre*^*,* so that hM3Dq-DREADD (Gq-DREADD) was expressed in *Agrp*^*Cre*^ cells. At P2, both control (*Agrp*^*Cre*^ or *Rosa*^*LSL-hM3Dq*^) and mutant (*Agrp*^*Cre*^*;Rosa*^*LSL-hM3Dq*^, termed *Agrp*^*hM3Dq*^) mice received a subcutaneous MSG injection to eliminate hypothalamic AgRP neurons (ARC-lesioned). At 8 weeks of age, ARC-lesioned control and *Agrp*^*hM3Dq*^ mice were injected with saline; food intake was measured for the next 3 h, and this treatment was repeated for 3 days. On the 4th day, both control and experimental mice were given a single injection of CNO and food intake was measured immediately afterward (Figure 3B). Both groups of mice had similar food intake upon saline injection. However, CNO injection led to significant increase of food intake and weight gain in the *Agrp*^*hM3Dq*^ mice but not in the control mice (Figure 3C). To evaluate if energy expenditure was also affected, we repeated the experiment by administering saline and CNO to control and mutant mice of similar body weights (45.14 ± 3.54 g and 41.83 ± 2.91 g, respectively) while in the CLAMS chambers. When compared with control mice, administration with CNO led to increased food intake and respiratory exchange ratio (RER) in *Agrp*^*hM3Dq*^ mice without altering energy expenditure (Figure 3D).

Upon CNO administration, FOS was induced in the ARC of *Agrp*^*hM3Dq*^ mice (ARC-intact) but not in *Agrp*^*hM3Dq*^ mice that were treated with MSG as neonates (ARC-lesioned), confirming ablation of AgRP^ARC^ neurons with neonatal MSG treatment (Figure 3E–F). FOS was induced in the AP and NTS of the ARC-lesioned *Agrp*^*hM3Dq*^ mice but not in control mice (Figure 3E–F). Together, these results suggest that chemogenetic activation of AgRP^Hind^ cells stimulated feeding in mice that lacked the AgRP^ARC^ neurons.

### 3.6 Focal transcranial optogenetic activation of hindbrain AgRP cells with SOUL opsin stimulates feeding

Finally, we determined if direct and focal activation of AgRP cells in the hindbrain would induce a hyperphagic response. We employed a minimally invasive optogenetic approach that utilizes a step-function opsin with ultra-high light sensitivity (SOUL), allowing minimally invasive transcranial light stimulation [16]. SOUL is a modified channelrhodopsin that has step function-like properties with extended channel open state and enhanced photocurrent amplitudes [16]. Illumination of SOUL-expressing cells with blue light (∼473 nm) leads to stable step in membrane potential (depolarization) in these cells and the channel can remain in the conducting state for more than 30 min [16].

**Figure 4:**
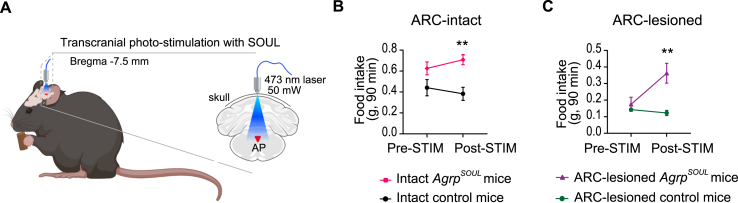
Transcranial activation of AgRP cells in the hindbrain promotes feeding. (**A**) Focal transcranial photo-stimulation of AgRP^Hind^ cells in mice that expressed the SOUL opsin in AgRP cells (*Agrp*^*Cre*^*;Rosa*^*LSL-SOUL*^) or in control mice (*Agrp*^*Cre*^ or *Rosa*^*LSL-SOUL*^). Fiber optic cannula was placed above the AP at Bregma −7.5. (**B**) Food intake was measured at the onset of dark cycle (7 PM) in *ad-lib* fed ARC-intact male mice (n = 9–10 per group) before and after photo-stimulation (STIM). (**C**) Food intake was measured at the onset of dark cycle (7 PM) in *ad-lib* fed 4-month-old ARC-lesioned male mice (n = 4 per group) before and after photo-stimulation. Data are mean ± SEM. ∗∗p < 0.01, by 2-WAY ANOVA with repeated measures and multiple comparisons between control and mutant mice.

To this end, we crossed *Agrp*^*Cre*^ mice with mice carrying *Rosa*^*LSL-SOUL*^, which directed expression of SOUL opsin in *Agrp*^*Cre*^ cells (these mice are termed *Agrp*^*SOUL*^). Mice carrying either *Agrp*^*Cre*^ or *Rosa*^*LSL-SOUL*^ were used as control. Adult *Agrp*^*SOUL*^ and control mice were affixed with a flat-end cannula on the surface of the intact skull above the AP area (Bregma −7.5 mm) (Figure 4A). In the absence of photo-stimulation, these mice consumed similar amount of food in the early phase of the dark cycle. In contrast, photo-stimulation of hindbrain AgRP neurons induced hyperphagia in *Agrp*^*SOUL*^ mice but not in control mice (Figure 4B).

As neurons in the hindbrain propagate neural signals to other brain regions including the hypothalamus, we further determined if orexigenic effects of activating hindbrain AgRP neurons may require the functions of ARC AgRP neurons. *Agrp*^*SOUL*^ mice were treated with MSG as neonates to ablate ARC AgRP neurons. At 10-weeks of age, ARC-lesioned *Agrp*^*SOUL*^ or control mice were subjected to focal transcranial optogenetic stimulation above the AP area. Photo-stimulation of hindbrain AgRP cells led to marked stimulation of food intake (Figure 4C). These findings corroborated the results from the chemogenetics experiments, demonstrating that hindbrain AgRP cells promote feeding independently of ARC AgRP neurons.

## 4 Discussion

AgRP neurons in the hypothalamus integrate diverse metabolic signals and environmental cues to coordinate feeding behaviors [14]. In this study, we identified a previously unknown population of AgRP cells in the mouse hindbrain, and demonstrated their orexigenic function.

Our results show that optogenetic activation of AgRP^Hind^ cells stimulates feeding in intact mice as well as in mice lacking AgRP^ARC^ neurons, suggesting that AgRP^Hind^ can regulate feeding independently of AgRP^ARC^ neurons. AgRP^ARC^ neurons are known to project to several hindbrain regions such as PAG and PBN, but AgRP^ARC^ neurons are not known to project to the AP or NTS (for review, see [14]). In support of this, an unbiased viral tracing approach reveals all known projection sites from AgRP^ARC^ neurons, but no projections to the AP/NTS region were observed [13]. It has been previously shown that activating ∼100 or less AgRP^ARC^ neurons is not sufficient to induce feeding [4], and that direct activation of AgRP^ARC^ projections to PAG and PBN does not induce feeding [36]. Moreover, MSG-treated mice, where majority of the AgRP^ARC^ neurons had been ablated, showed even greater hyperphagia compared with mice with intact AgRP^ARC^ neurons (Figure 4B–C and Fig. S7). Thus, it is unlikely that residue projections to the hindbrain from remaining AgRP^ARC^ neurons are responsible for the hyperphagic response induced by stimulating AgRP^Hind^ cells.

Recent studies have shown that ARC-projecting TH^+^ catecholamine neurons in the NTS mediate hypoglycemia-induced feeding [37] and that TH^+^/NPY^+^ NTS neurons stimulate feeding [38]. Majority of AgRP^Hind^ cells do not express *Th* or *Npy* (Table S1). Moreover, while AgRP^ARC^ neuronal activity is required for glucoprivic feeding induced by NTS TH ^+^ neurons [37], our data indicate that feeding induced by AgRP^Hind^ cells were independent of AgRP^ARC^ neurons as the orexigenic effects are preserved and even heightened in MSG-treated mice (Figure 4B–C and Fig. S7). These observations suggest that AgRP^Hind^ cells represent a separate population of cells distinct from the NTS TH ^+^ neurons. A unique characteristic of the hindbrain AgRP cells is that they consist of both neurons and tanycyte-like cells in postnatal mice. Tanycytes are best defined as the specialized, radial glial-like ependymal cells lining the 3rd ventricle of the hypothalamus [39]. Tanycytes have progenitor cell properties and can give rise to ependymal cells, tanycytes, astrocytes and neurons [[40], [41], [42]]. Indeed, hypothalamic tanycytes have the capacity to differentiate into neurons [40,41,43], and cells resembling tanycytes are present in the AP [30,44,45]. CNS neuronal circuits exhibit incredible plasticity, especially during the embryonic and early postnatal period, so that animals can adapt to a new environment. Future experiments will be required to assess the roles of AgRP^+^ tanycytes.

It is intriguing to note that AgRP^ARC^ and AgRP^Hind^ cells are situated in or near the median eminence and the area postrema, respectively, circumventricular organs that lack an intact BBB. We have previously shown that more than half of the AgRP^ARC^ are outside the BBB, allowing these cells to sense dynamic changes of circulating metabolic signals [27,46]. Thus, like their hypothalamic counterparts, AgRP cells in the AP are in open communication with the circulation and could be key sensors of circulating nutrients and hormonal signals. The AP and NTS are prime integration sites of circulating hormonal signals as well as ascending neuronal input from the GI tract. It is interesting to note that a subset of hindbrain AgRP cells in the adult mice expressed GLP-1 receptor, calcitonin receptor, insulin receptor and glucocorticoid receptor (Table S1). Whether hindbrain AgRP cells are suppressed by GLP-1, amylin, or insulin remain to be determined. It is worth mentioning that optogenetic activation of AgRP^Hind^ cells yielded hyperphagic response in nighttime (Figure 4B–C) but not in daytime (Fig. S9). The mechanism behind this diurnal effect requires further determination. It is possible that a critical component of the hindbrain AgRP cells is under circadian regulation, enabling its greater release during early dark cycle. Alternatively, activation of AgRP^Hind^ cells may simply instruct a permissive condition to feed, but actual feeding may also require other enabling signals present in the dark cycle.

The role of AgRP in feeding regulation is unsettled as AgRP-deficient mice have normal feeding and body weight [47]. Intriguingly, GABA and NPY, components of the AgRP^ARC^ neurons, mediate the fast-feeding response whereas AgRP mediates a delayed but prolonged response upon acute activation of AgRP^ARC^ neurons [48]. Given that AgRP expression is very low in the hindbrain under normal conditions, the rapid hyperphagic effects induced by activating hindbrain AgRP cells could be mediated through the release of small neurotransmitters from AgRP^Hind^ neurons. In contrast to the AgRP^ARC^ neurons which are mostly GABAergic, hindbrain AgRP neurons are a mix of GABAergic and glutamatergic neurons. Thus, it seems that hindbrain AgRP cells are heterogeneous in nature, both in their anatomical location within the DMV (AP, SubP or NTS) and likely their connectivity to different target neurons. The existence of distinct subcircuits engaged by different subpopulations of AgRP neurons could afford extra levels of flexibility in feeding regulation. Of note, the central melanocortin system in the hindbrain has been implicated to play a role in feeding. For example, 4th ventricle infusion of α-MSH inhibits feeding, whereas infusion of an melanocortin receptor antagonist SHU9119 stimulates feeding in a dose-dependent manner [49]. Acute activation of POMC^NTS^ neurons leads to immediate inhibition of feeding whereas chronic activation of POMC^ARC^ neurons is required to suppress food intake [12], highlighting the role of POMC^NTS^ neurons in regulation of food intake. Within the DVC, *Mc4r* is most abundantly expressed in the DMV, and MC4R neurons in the DMV regulate autonomic functions [7], energy expenditure but not feeding [8]. Our observation that activation of AgRP^Hind^ cells affects feeding but not energy expenditure suggests that these effects may not be mediated by MC4R in the DMV. The increase in RER caused by activation of AgRP^Hind^ cells is likely secondary to increased food intake on a chow diet that is high in carbohydrate content. Interestingly, *Mc4r*^*+*^ cells were detected in the AP after treatment with GLP1 agonist [45]. By immunofluorescence analysis using *Mc4r*^*Cre*^*; Rosa*^*LSL-tDT*^ mice [20], we detected a population of tdTomato^+^ cells in the AP and more abundantly in the SubP (Fig. S8). The functions of MC4R neurons in the AP and SubP have not been characterized, but their close proximity to the AgRP^Hind^ neurons indicates that AgRP released from AgRP^Hind^ neurons could target this subpopulation of MC4R neurons. Notably, expression of AgRP in hindbrain cells and their neuronal activity increase upon food deprivation, suggesting that these cells can act as sensors for energy deficit. Maintenance of orexigenic drive is essential for survival. As such, having neuronal circuits that have redundant or overlapping functions could safeguard feeding when one of the circuits is functionally compromised. Consistent with this notion, activating AgRP^Hind^ neurons in mice lacking AgRP^ARC^ neurons elicits greater increase of food intake compared to mice with intact AgRP^ARC^ neurons.

In summary, this study delineates the existence and function of a previously unknown population of AgRP cells in the mouse hindbrain that drives feeding. This study may pave the way for future anti-obesity therapeutic interventions aiming to silence AgRP neurons in the area postrema, an area outside the BBB that enables easy access of therapeutic agents.

## CRediT authorship contribution statement

**Tomas P. Bachor:** Conceptualization, Data curation, Formal analysis, Investigation, Methodology, Writing – original draft, Writing – review & editing, Funding acquisition. **Eunsang Hwang:** Data curation, Formal analysis, Investigation, Methodology, Writing – original draft, Writing – review & editing. **Ernie Yulyaningsih:** Conceptualization, Data curation, Formal analysis, Funding acquisition, Investigation, Methodology. **Kush Attal:** Data curation, Formal analysis. **Francois Mifsud:** Data curation, Formal analysis. **Viana Pham:** Data curation, Formal analysis. **Eirini Vagena:** Data curation, Formal analysis. **Renzo Huarcaya:** Data curation, Formal analysis. **Martin Valdearcos:** Data curation, Formal analysis. **Christian Vaisse:** Data curation, Formal analysis, Writing – review & editing. **Kevin W. Williams:** Data curation, Formal analysis, Investigation, Methodology, Supervision, Writing – original draft, Writing – review & editing. **Paul J. Emmerson:** Funding acquisition, Supervision, Writing – review & editing. **Allison W. Xu:** Conceptualization, Funding acquisition, Project administration, Supervision, Writing – original draft, Writing – review & editing.

## Declaration of competing interest

The authors declare that they have no known competing financial interests or personal relationships that could have appeared to influence the work reported in this paper.

## Data Availability

Single Cell RNAseq data from this study are deposited in the NCBI GEO database (GEO accession number GSE204865). No original code was developed in this study.

## References

[1] Yeo G.S.H., Chao D.H.M., Siegert A.M., Koerperich Z.M., Ericson M.D., Simonds S.E. (2021). The melanocortin pathway and energy homeostasis: from discovery to obesity therapy. Mol Metabol.

[2] Ollmann M.M., Wilson B.D., Yang Y.K., Kerns J.A., Chen Y., Gantz I. (1997). Antagonism of central melanocortin receptors in vitro and in vivo by agouti-related protein. Science.

[3] Rossi M., Kim M.S., Morgan D.G., Small C.J., Edwards C.M., Sunter D. (1998). A C-terminal fragment of Agouti-related protein increases feeding and antagonizes the effect of alpha-melanocyte stimulating hormone in vivo. Endocrinology.

[4] Aponte Y., Atasoy D., Sternson S.M. (2011). AGRP neurons are sufficient to orchestrate feeding behavior rapidly and without training. Nat Neurosci.

[5] Krashes M.J., Koda S., Ye C., Rogan S.C., Adams A.C., Cusher D.S. (2011). Rapid, reversible activation of AgRP neurons drives feeding behavior in mice. J Clin Invest.

[6] Grill H.J., Hayes M.R. (2012). Hindbrain neurons as an essential hub in the neuroanatomically distributed control of energy balance. Cell Metabol.

[7] Sohn J.W., Harris L.E., Berglund E.D., Liu T., Vong L., Lowell B.B. (2013). Melanocortin 4 receptors reciprocally regulate sympathetic and parasympathetic preganglionic neurons. Cell.

[8] Rossi J., Balthasar N., Olson D., Scott M., Berglund E., Lee C.E. (2011). Melanocortin-4 receptors expressed by cholinergic neurons regulate energy balance and glucose homeostasis. Cell Metabol.

[9] Georgescu T., Lyons D., Doslikova B., Garcia A.P., Marston O., Burke L.K. (2020). Neurochemical characterization of brainstem pro-opiomelanocortin cells. Endocrinology.

[10] Zheng H., Patterson L.M., Phifer C.B., Berthoud H.R. (2005). Brain stem melanocortinergic modulation of meal size and identification of hypothalamic POMC projections. Am J Physiol Regul Integr Comp Physiol.

[11] Butler A.A., Cone R.D. (2003). Knockout studies defining different roles for melanocortin receptors in energy homeostasis. Ann N Y Acad Sci.

[12] Zhan C., Zhou J., Feng Q., Zhang J.E., Lin S., Bao J. (2013). Acute and long-term suppression of feeding behavior by POMC neurons in the brainstem and hypothalamus, respectively. J Neurosci.

[13] Wang D., He X., Zhao Z., Feng Q., Lin R., Sun Y. (2015). Whole-brain mapping of the direct inputs and axonal projections of POMC and AgRP neurons. Front Neuroanat.

[14] Deem J.D., Faber C.L., Morton G.J. (2022). AgRP neurons: regulators of feeding, energy expenditure, and behavior. FEBS J.

[15] Maier M.T., Vilhelmsson A., Louie S.M., Vagena E., Nomura D.K., Koliwad S.K. (2018). Regulation of hepatic lipid accumulation and distribution by agouti-related protein in male mice. Endocrinology.

[16] Gong X., Mendoza-Halliday D., Ting J.T., Kaiser T., Sun X., Bastos A.M. (2020). An ultra-sensitive step-function opsin for minimally invasive optogenetic stimulation in mice and macaques. Neuron.

[17] Wang Q., Liu C., Uchida A., Chuang J.C., Walker A., Liu T. (2014). Arcuate AgRP neurons mediate orexigenic and glucoregulatory actions of ghrelin. Mol Metabol.

[18] Tong Q., Ye C.P., Jones J.E., Elmquist J.K., Lowell B.B. (2008). Synaptic release of GABA by AgRP neurons is required for normal regulation of energy balance. Nat Neurosci.

[19] Zhu H., Aryal D.K., Olsen R.H., Urban D.J., Swearingen A., Forbes S. (2016). Cre-dependent DREADD (designer receptors exclusively activated by designer Drugs) mice. Genesis.

[20] Garfield A.S., Li C., Madara J.C., Shah B.P., Webber E., Steger J.S. (2015). A neural basis for melanocortin-4 receptor-regulated appetite. Nat Neurosci.

[21] Madisen L., Zwingman T.A., Sunkin S.M., Oh S.W., Zariwala H.A., Gu H. (2010). A robust and high-throughput Cre reporting and characterization system for the whole mouse brain. Nat Neurosci.

[22] Saxena A., Wagatsuma A., Noro Y., Kuji T., Asaka-Oba A., Watahiki A. (2012). Trehalose-enhanced isolation of neuronal sub-types from adult mouse brain. Biotechniques.

[23] Stuart T., Butler A., Hoffman P., Hafemeister C., Papalexi E., Mauck W.M. (2019). Comprehensive integration of single-cell data. Cell.

[24] Dong Y., Carty J., Goldstein N., He Z., Hwang E., Chau D. (2021). Time and metabolic state-dependent effects of GLP-1R agonists on NPY/AgRP and POMC neuronal activity in vivo. Mol Metabol.

[25] He Z., Gao Y., Lieu L., Afrin S., Cao J., Michael N.J. (2019). Direct and indirect effects of liraglutide on hypothalamic POMC and NPY/AgRP neurons - implications for energy balance and glucose control. Mol Metabol.

[26] Hwang E., Scarlett J.M., Baquero A.F., Bennett C.M., Dong Y., Chau D. (2022). Sustained inhibition of NPY/AgRP neuronal activity by FGF1. JCI Insight.

[27] Yulyaningsih E., Rudenko I.A., Valdearcos M., Dahlen E., Vagena E., Chan A. (2017). Acute lesioning and rapid repair of hypothalamic neurons outside the blood-brain barrier. Cell Rep.

[28] Nelson N.G., Wu L., Maier M.T., Lam D., Cheang R., Alba D. (2022). A gene-diet interaction controlling relative intake of dietary carbohydrates and fats. Mol Metabol.

[29] Vagena E., Crneta J., Engstrom P., He L., Yulyaningsih E., Korpel N.L. (2022). ASB4 modulates central melanocortinergic neurons and calcitonin signaling to control satiety and glucose homeostasis. Sci Signal.

[30] Zhang C., Kaye J.A., Cai Z., Wang Y., Prescott S.L., Liberles S.D. (2021). Area postrema cell types that mediate nausea-associated behaviors. Neuron.

[31] Wilson B.D., Bagnol D., Kaelin C.B., Ollmann M.M., Gantz I., Watson S.J. (1999). Physiological and anatomical circuitry between Agouti-related protein and leptin signaling. Endocrinology.

[32] Burde R.M., Schainker B., Kayes J. (1971). Acute effect of oral and subcutaneous administration of monosodium glutamate on the arcuate nucleus of the hypothalamus in mice and rats. Nature.

[33] Olney J.W., Sharpe L.G. (1969). Brain lesions in an infant rhesus monkey treated with monsodium glutamate. Science.

[34] Olney J.W. (1969). Brain lesions, obesity, and other disturbances in mice treated with monosodium glutamate. Science.

[35] Broberger C., Johansen J., Johansson C., Schalling M., Hokfelt T. (1998). The neuropeptide Y/agouti gene-related protein (AGRP) brain circuitry in normal, anorectic, and monosodium glutamate-treated mice. Proc Natl Acad Sci U S A.

[36] Betley J.N., Cao Z.F., Ritola K.D., Sternson S.M. (2013). Parallel, redundant circuit organization for homeostatic control of feeding behavior. Cell.

[37] Aklan I., Sayar Atasoy N., Yavuz Y., Ates T., Coban I., Koksalar F. (2020). NTS catecholamine neurons mediate hypoglycemic hunger via medial hypothalamic feeding pathways. Cell Metabol.

[38] Chen J., Cheng M., Wang L., Zhang L., Xu D., Cao P. (2020). A vagal-NTS neural pathway that stimulates feeding. Curr Biol.

[39] Rodriguez E.M., Blazquez J.L., Pastor F.E., Pelaez B., Pena P., Peruzzo B. (2005). Hypothalamic tanycytes: a key component of brain-endocrine interaction. Int Rev Cytol.

[40] Lee D.A., Bedont J.L., Pak T., Wang H., Song J., Miranda-Angulo A. (2012). Tanycytes of the hypothalamic median eminence form a diet-responsive neurogenic niche. Nat Neurosci.

[41] Haan N., Goodman T., Najdi-Samiei A., Stratford C.M., Rice R., El Agha E. (2013). Fgf10-expressing tanycytes add new neurons to the appetite/energy-balance regulating centers of the postnatal and adult hypothalamus. J Neurosci.

[42] Robins S.C., Stewart I., McNay D.E., Taylor V., Giachino C., Goetz M. (2013). alpha-Tanycytes of the adult hypothalamic third ventricle include distinct populations of FGF-responsive neural progenitors. Nat Commun.

[43] Surbhi, Wittmann G., Low M.J., Lechan R.M. (2021). Adult-born proopiomelanocortin neurons derived from Rax-expressing precursors mitigate the metabolic effects of congenital hypothalamic proopiomelanocortin deficiency. Mol Metabol.

[44] Langlet F., Mullier A., Bouret S.G., Prevot V., Dehouck B. (2013). Tanycyte-like cells form a blood-cerebrospinal fluid barrier in the circumventricular organs of the mouse brain. J Comp Neurol.

[45] Ludwig M.Q., Cheng W., Gordian D., Lee J., Paulsen S.J., Hansen S.N. (2021). A genetic map of the mouse dorsal vagal complex and its role in obesity. Nat Metab.

[46] Olofsson L.E., Unger E.K., Cheung C.C., Xu A.W. (2013). Modulation of AgRP-neuronal function by SOCS3 as an initiating event in diet-induced hypothalamic leptin resistance. Proc Natl Acad Sci U S A.

[47] Qian S., Chen H., Weingarth D., Trumbauer M.E., Novi D.E., Guan X. (2002). Neither agouti-related protein nor neuropeptide Y is critically required for the regulation of energy homeostasis in mice. Mol Cell Biol.

[48] Krashes M.J., Shah B.P., Koda S., Lowell B.B. (2013). Rapid versus delayed stimulation of feeding by the endogenously released AgRP neuron mediators GABA, NPY, and AgRP. Cell Metabol.

[49] Grill H.J., Ginsberg A.B., Seeley R.J., Kaplan J.M. (1998). Brainstem application of melanocortin receptor ligands produces long-lasting effects on feeding and body weight. J Neurosci.

