# Supplementary material for: Identification of AgRP cells in the murine hindbrain that drive feeding
Source: Mol Metab. 2024 Jan 19;80:101886. doi: 10.1016/j.molmet.2024.101886 (PMC10844855; doi:10.1016/j.molmet.2024.101886)
Supplement: Multimedia component 2 [file mmc2.pdf]

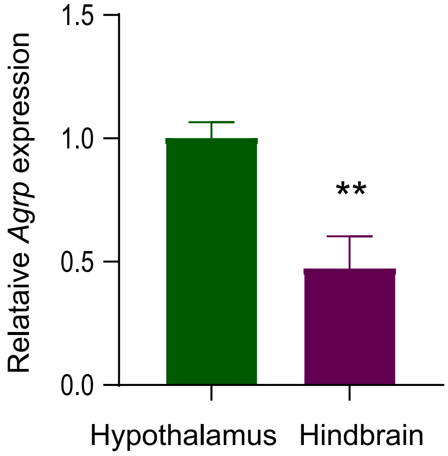


**Supplementary Figure 1: Comparison of Agrp mRNA expression in fed and fasted conditions in hypothalamic and hindbrain tissue.**
Brains were dissected from 10-week-old female mice (n=5) under *ad-libitum* fed condition, and specific regions containing the hypothalamus or hindbrain tissues were processed for qRT-PCR using an *Agrp* TaqMan™ probe. *Agrp* mRNA values were normalized to levels in the hypothalamus**.** ** $p<0.01$ by student's t test.

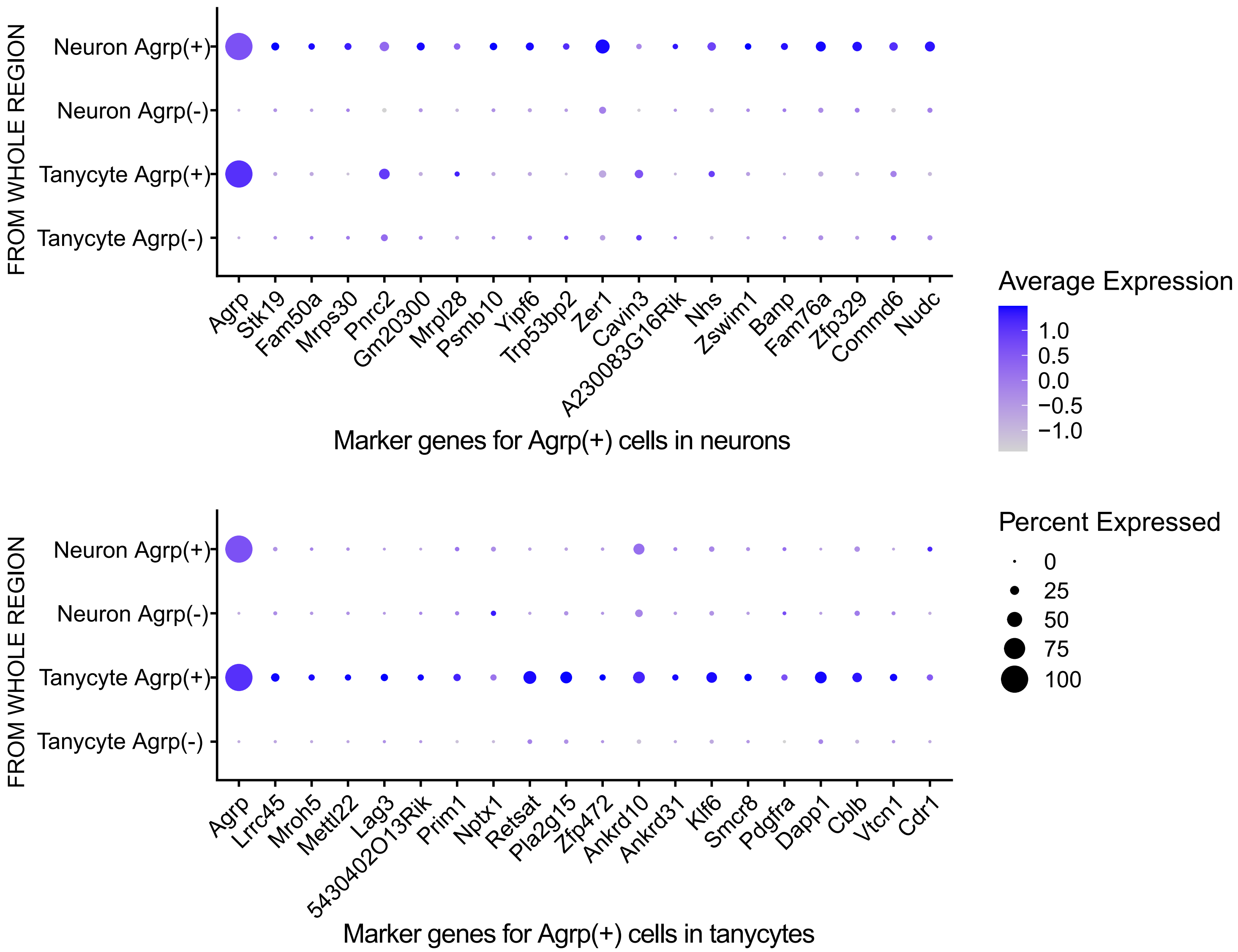


**Supplementary Figure 2. Most specific marker genes of *Agrp*-expressing neurons and tanycytes in the hindbrain single-nuclei dataset.** The hindbrain single-nuclei dataset (Geo accession number GSE160938) (Zhang C et al. 2021. Neuron 109: 461-472) was re-analyzed to find the most specific gene markers of Agrp-expressing cells in the hindbrain, both for neurons and tanycytes. Briefly, the publicly available raw z-umi count matrixes were downloaded from the authors' GitHub repository. The R package Seurat v4.1.0 was used to process single nuclei RNA-seq count data. Quality control, filtering, normalization and cell type based clustering was performed as in the original publication. *Agrp*-expressing nuclei were defined as nuclei in which an *Agrp* transcript was detected (RNA-seq count greater than zero). Differential expression analysis between *Agrp* expressing cells and non-*Agrp* expressing cells was conducted using Seurat's FindMarkers() function, separately in the neurons cluster and in the tanycytes cluster. The top 20 marker genes for each cell type were plotted with Seurat's Dotplot() function.

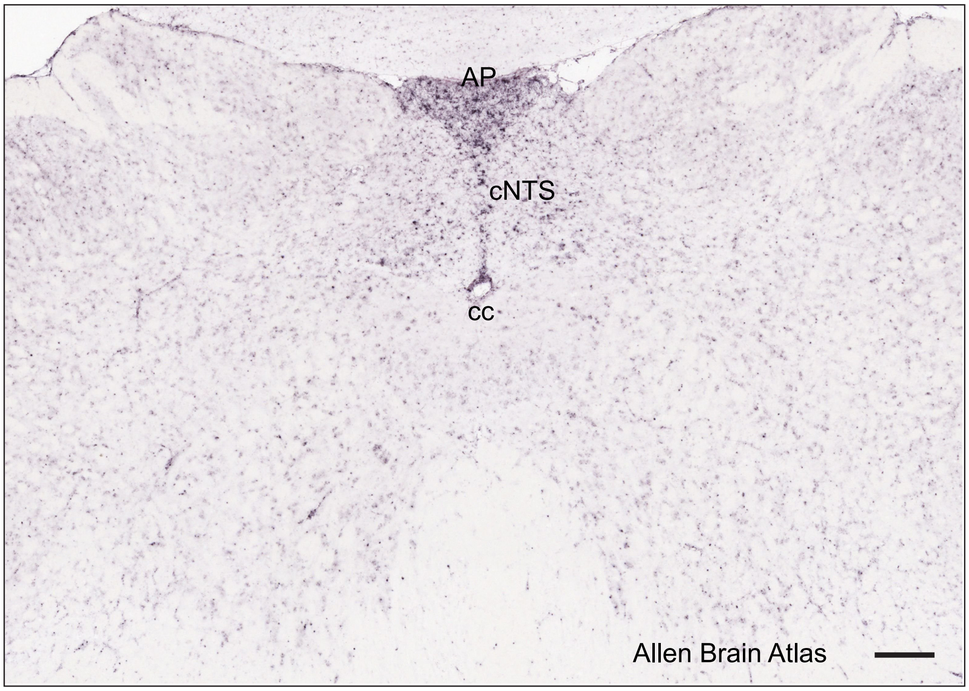


**Supplementary Figure 3: Expression of tdTomato mRNA in the hindbrain region.** Image is cited from Allen Brain Atlas showing mRNA in situ hybridization against tdTomato in a 77-day-old mouse with Agrp-IRES-Cre;Ai14(RCL-tdT) - RP_090804_03_H02. AP: area postrema. cNTS: commissural nucleus of the solitary tract. cc: central canal. Scale bar: 200 μm.

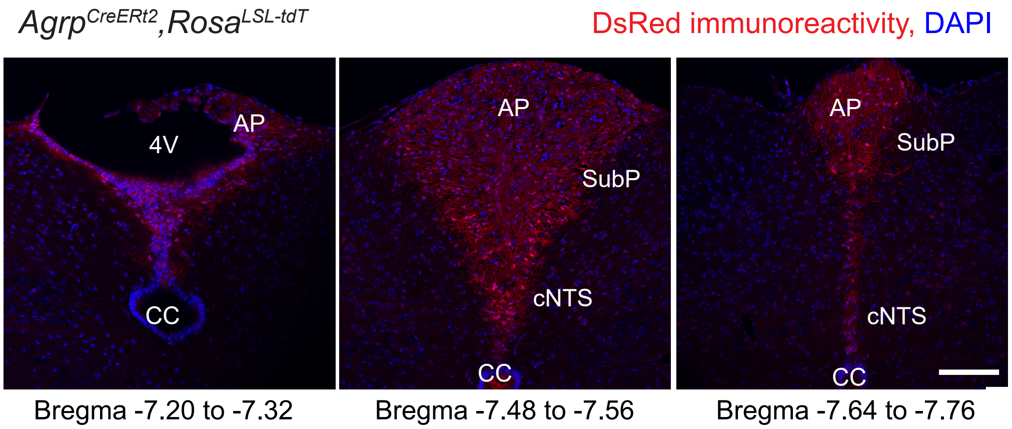


**Supplementary Figure 4.** ***Agrp$^{CreERt2}$*** **transgenic mice direct reporter expression in the hindbrain.**

Immunofluorescence analysis using DsRed antibody showing tdTomato expression from rostral to caudal AP in an *Agrp$^{CreERt2}$*, *Rosa$^{LSL\text{-}tdT}$* mouse that was injected with tamoxifen at 4 weeks of age. Scale bar: 100 µm. AP: area postrema. SubP: subpostrema area. cNTS: commissural nucleus of solitary track. 4V: fourth ventricle. cc: central canal.

*Agrp*$^{Cre}$,*Rosa*$^{LSL\text{-}tdT}$ (tdT, Nestin, DAPI)

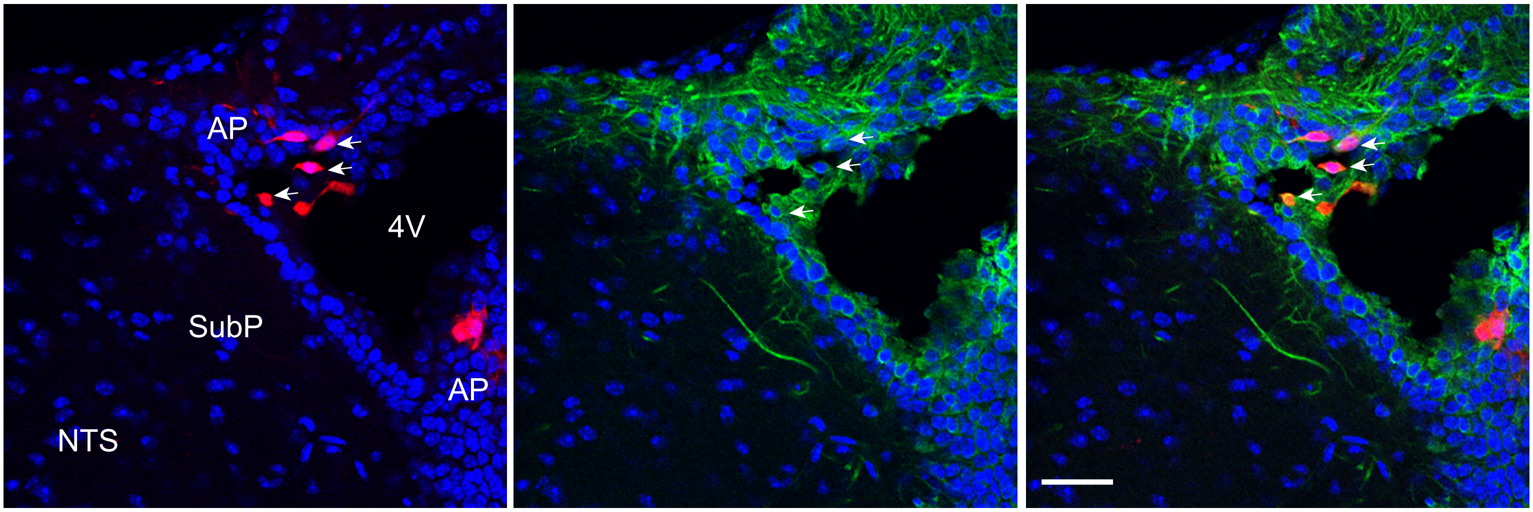


Bregma -7.20 to -7.32

**Supplementary Figure 5. Immunofluorescence analysis of Nestin expression in the DVC of Agrp$^{Cre}$,Rosa$^{LSL\text{-}tdT}$ mice.**
The brains of 8 week old male *Agrp*$^{Cre}$,*Rosa*$^{LSL\text{-}tdT}$ mice (n=3) were subjected to immunofluorescence using antibodies against tdTomato and Nestin and imaged with a confocal microscope in single plane mode. Strong tdTomato immunoreactivity is shown in the rostral AP region. White arrows indicate co-localization of tdTomato with Nestin expressing cells. Scale bar 50 µm. AP: area postrema. SubP: subpostrema area. NTS: commissural nucleus of solitary track. 4V: fourth ventricle.

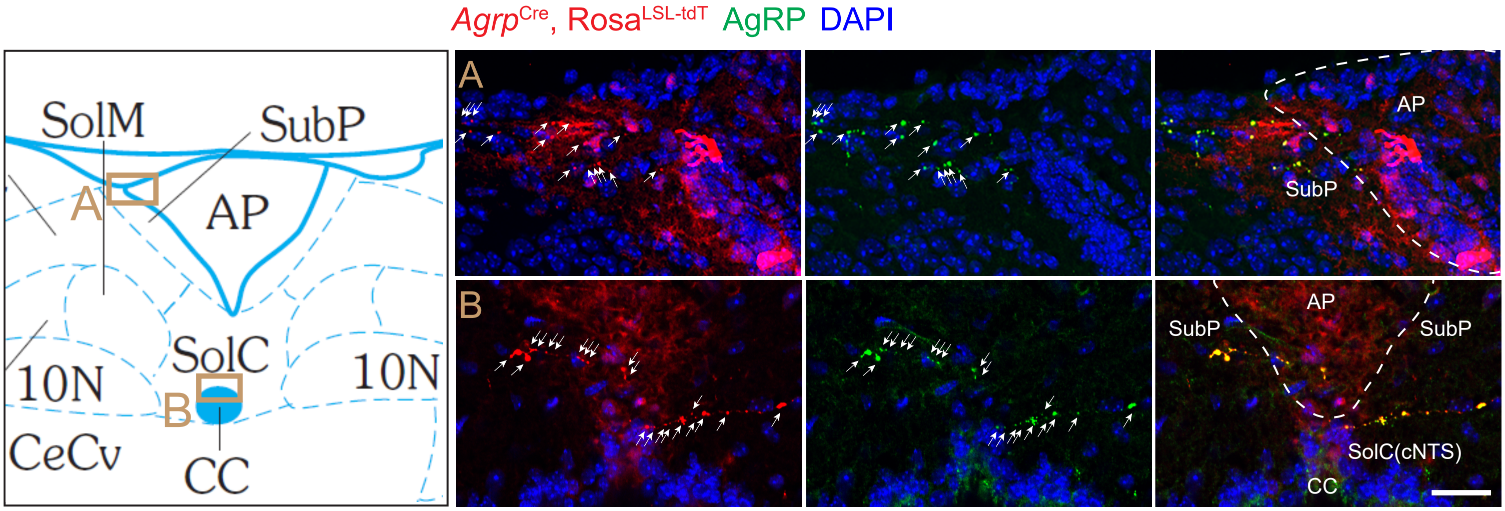


**Supplementary Figure 6. Partial colocalization of tdTomato and AgRP in neuronal boutons within SubP and cNTS.** Representative images showing presence of AgRP-positive and tdTomato-positive boutons in the AP, SubP and cNTS and their partial colocalization. Arrows indicate boutons that show overlapping AgRP and tdTomato expression. Scale bar: 50 µm. cc: central canal. AP: area postrema. SubP: subpostrema area. SolC/cNTS: solitary nucleus, commissural part. SolM: solitary nucleus, medial part. 10N: dorsal motor nucleus of vagus. Atlas is cited from “The Mouse Brain” by Franklin and Paxinos, third edition. The locations of the image fields in A and B are outlined in boxes with labels on the atlas.

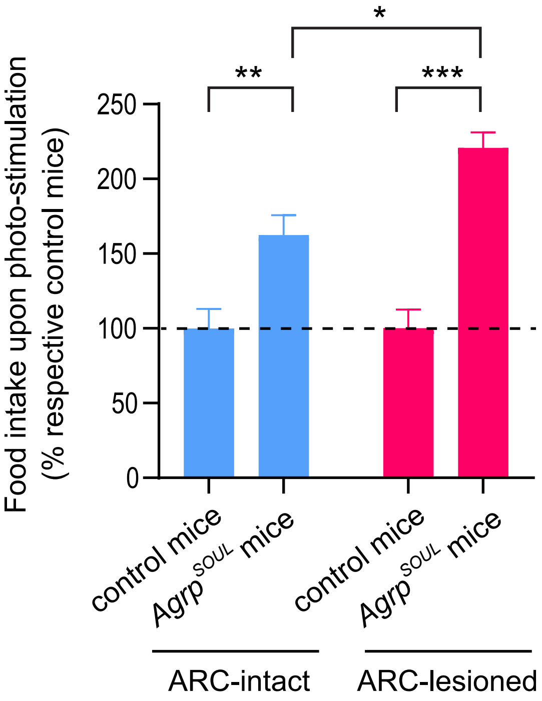


**Supplementary Figure 7: Hyperphagic effects induced by activating hindbrain AgRP cells in mice with intact or lesioned arcuate nucleus.** Focal transcranial photo-stimulation of $\mathrm{AgRP}^{\mathrm{Hind}}$ cells in mice that expressed the SOUL opsin in AgRP cells ($Agrp^{Cre};Rosa^{LSL\text{-}SOUL}$). Control mice carried either $Agrp^{Cre}$ or $Rosa^{LSL\text{-}SOUL}$. Control and experimental mice had either intact arcuate nucleus (n=9-10/group) or were treated with MSG during the neonatal period (n=4/group). Food intake was measured at the onset of dark cycle (7 PM). This figure is another representation of data in Figure 4, but with food intake of experimental mice normalized to that of their respective control mice. * $p<0.05$, ** $p<0.01$, *** $p<0.001$ by 2-way ANOVA with Sidak multiple comparison test.

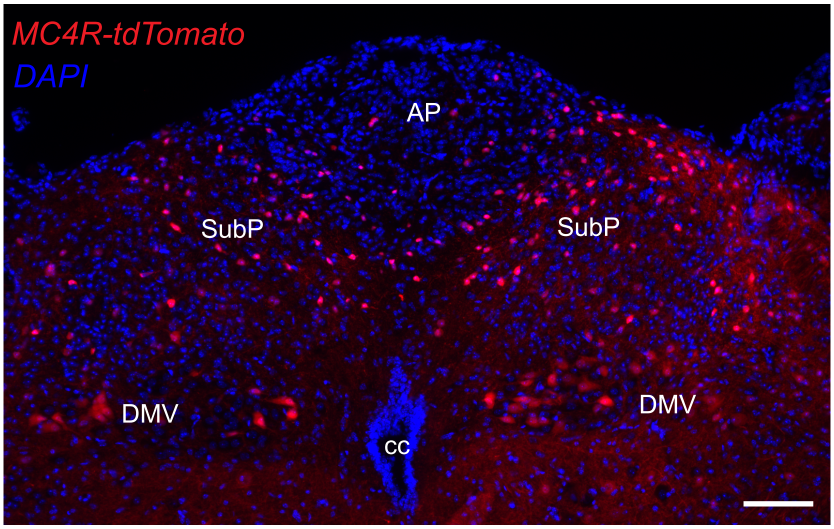


**Supplementary Figure 8: Expression of *Mc4r-tdTomato* in the AP and SubP.** Immunofluorescence analysis with DsRed antibody showing tdTomato expression in adult $\textit{Mc4r}^{Cre}$, $\textit{Rosa}^{LSL\text{-}tdT}$ mice. AP: area postrema, SubP: subpostrema area, DMV: dorsal motor nucleus of the vagus. cc: central canal. Scale bar: 100 μm.

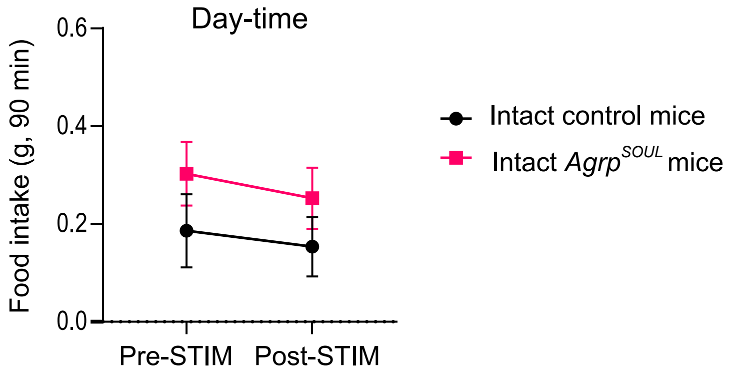


**Supplementary Figure 9: Daytime food intake measurements before and after optogenetic stimulation.**
Focal transcranial photo-stimulation of AgRP$^{Hind}$ cells in mice that expressed the SOUL opsin in AgRP cells (*Agrp*$^{Cre}$;*Rosa*$^{LSL\text{-}SOUL}$). Control mice carried either *Agrp*$^{Cre}$ or *Rosa*$^{LSL\text{-}SOUL}$. Fiber optic cannula was placed above the AP at Bregma -7.5. Food intake was measured at daytime (12 PM) in *ad-lib* fed ARC-intact male mice (n=9-10, per group) before and after photo-stimulation (STIM).
